# Reduced Alpha Power Associated with Impaired Dual-Task Standing Performance in Older Adults with MCI

**DOI:** 10.1093/geroni/igaf122.3366

**Published:** 2025-12-31

**Authors:** Helia Osareh, Brad Manor, Melike Kahya

**Affiliations:** High Point University, High Point, North Carolina, United States; Hebrew SeniorLife/Harvard Medical School, Boston, Massachusetts, United States; High Point University, High Point, North Carolina, United States

## Abstract

Older adults, particularly those with mild cognitive impairment (MCI), often struggle with standing balance during dual-tasking. These difficulties arise from limited cognitive processing capacity, with MCI individuals showing greater dual-task cost than healthy older adults. While Electroencephalogram (EEG) studies have found that MCI is associated with reduced alpha power (8-13 Hz), the influence of dual-tasking on brainwave patterns and its relationship to dual-tasking standing performance remain unclear. We hypothesized that older adults with MCI would show reduced alpha power during dual-tasking compared to cognitively-intact controls, and that this reduction would also correlate with worse dual-tasking performance. We recruited 15 cognitive-intact participants (Montreal Cognitive Assessment (MoCA) score = 25-30) and ten participants with MCI (MoCA score = 21-24). Participants completed standing balance assessments while performing verbal subtractions. EEG alpha, beta, gamma, and theta power were recorded throughout the task. APDM Opal sensors were used to measure postural sway metrics synchronously with EEG recording. Older adults with MCI, as compared to cognitively-intact controls, exhibited lower alpha power, particularly in the central right (p = .003) and anterior left (p = .002) regions of the brain. Beta, theta, and gamma brain waves were not significantly different between groups. Reduced alpha power during dual-tasking was associated with increased postural sway path in older adults with MCI (r = -0.47, p = 0.01). These results suggest that reduced alpha power is correlated with worse dual-task standing performance. Future research should explore whether reduced alpha power contributes to impaired dual-task standing performance in older adults with MCI.

